# Early discharge of term neonates: we can do it safely

**DOI:** 10.1186/1824-7288-41-S2-A42

**Published:** 2015-09-30

**Authors:** R Luciano

**Affiliations:** 1 Neonatology Unit, Gemelli University Hospital, 00168 Rome, Italy

## 

Discharge of the term newborn is a critical issue in perinatal care. The average length of stay of the mother-infant dyad after delivery declined steadily from 1970 until the mid-1990s (early discharge ≤ 48 hours, very early discharge ≤ 24 hours after birth) [1]. Several subsequent studies [2-5] have reported that too short a hospital stay can place an infant at risk for significant jaundice, feeding difficulties, hypernatraemic dehydration, undetected infections, ductal-dependant cardiac lesions or gastrointestinal obstruction and may result in readmission [6-8]. Stopping or not initiating breastfeeding due to a lack of support for breastfeeding practice [9] is also matter of concern, taking into account that the postnatal period can be a critical one for the mother (postpartum blues, family relations issues in the new family context). Moreover, postnatal care gaps may result from non-activation of local services for postnatal counseling, delays in the first visit after discharge at the birth center, or late takeover by the family pediatrician.

The recent pronouncements of scientific societies [10] and the World Health Organization (2013) [11] together with legislation produced in our country (Progetto Obiettivo Materno Infantile 2000, Piano Sanitario Nazionale 2006 - 2008, Conferenza Unificata Stato-Regioni 2010, Raccomandazione n° 16 del Ministero della Salute 2014), can help identify criteria for appropriate and safe discharge of the mother-infant dyad. All efforts should be made to promote simultaneous mother-neonate discharge and the length of hospital stay should be based on the unique characteristics of each mother-infant dyad, including not only the health of the mother and the neonate but also the ability and confidence of the mother to care for her infant, the adequacy of support systems at home, and the access to appropriate follow-up care (Table 1).

**Table 1:** Criteria to be met before discharge of a term neonate (modified from American Academy of Pediatrics 2015)

*A)*	*Neonatal health*
1.	Clinical course and physical examination at discharge have not revealed abnormalities that require continued hospitalization

2.	Infant's vital signs within normal ranges and stable for the 12 hours preceding discharge

3.	The infant has urinated regularly and passed at least 1 stool spontaneously

4.	The infant is able to coordinate sucking, swallowing, and breathing while feeding

5.	The clinical risk of development of subsequent hyperbilirubinemia has been assessed, and appropriate management and/or follow-up plans have been instituted as recommended in guidelines for management of hyperbilirubinemia

6.	The infant has been adequately evaluated and monitored for sepsis on the basis of maternal risk factors and in accordance with current guidelines for prevention of perinatal group B streptococcal disease.

7.	Availability and evaluation of maternal screening results for syphilis, hepatitis B, HIV and appropriate treatment instituted when needed

8.	Newborn metabolic and hearing screenings completed

***B)***	***Maternal competency***

1.	Breastfeeding (positions, latch-on, efficacy of swallowing, importance and benefits) or bottle feeding

2.	Appropriate urination and defecation frequency for the infant

3.	Cord, skin, and genital care for the infant

4.	Infant safety

5.	The ability to recognize signs of illness and common infant problems, particularly jaundice

***C)***	***Assessment of family, environmental, and social risk factors and discussions with social services when plan to safeguard the infant is needed***

1.	Untreated parental substance abuse or positive urine toxicology results in the mother or newborn

2.	History of child abuse or neglect or history of domestic violence

3.	Mental illness in a parent who is in the home

4.	Lack of social support, particularly for adolescent mother or single mother who live in a shelter, a rehabilitation home, or on the street

5.	Communicable illness in a parent or other members of the household

6.	Assessment of barriers to adequate follow-up care for the newborn, such as lack of transportation to medical care services or language barriers to make suitable arrangements to address the family

***D)***	***Plan for continuing medical care***

1.	Identification of medical services for postnatal checks

2.	Date of first appointment after discharge

3.	Planning bilirubin check or other individualized controls when needed
